# Protex—A Python utility for proton exchange in molecular dynamics simulations

**DOI:** 10.3389/fchem.2023.1140896

**Published:** 2023-02-17

**Authors:** Florian Joerg, Marcus Wieder, Christian Schröder

**Affiliations:** ^1^ Department of Computational Biological Chemistry, Faculty of Chemistry, University of Vienna, Vienna, Austria; ^2^ Vienna Doctoral School in Chemistry (DoSChem), University of Vienna, Vienna, Austria; ^3^ Department of Pharmaceutical Sciences, Faculty of Life Sciences, University of Vienna, Vienna, Austria

**Keywords:** molecular dynamics, ionic liquids, dynamic properties, proton exchange, conductivity

## Abstract

Protex is an open-source program that enables proton exchanges of solvent molecules during molecular dynamics simulations. While conventional molecular dynamics simulations do not allow for bond breaking or formation, protex offers an easy-to-use interface to augment these simulations and define multiple proton sites for (de-)protonation using a single topology approach with two different *λ*-states. Protex was successfully applied to a protic ionic liquid system, where each molecule is prone to (de-)protonation. Transport properties were calculated and compared to experimental values and simulations without proton exchange.

## 1 Introduction

Molecular dynamics (MD) simulations have become indispensable in modern computational science. Over the last decades, major improvements have been made regarding the size and speed so that nowadays, biologically relevant systems [*i.e.*, membrane proteins (Goossens and De Winter 2018)] and many others can be studied in acceptable timescales (Hospital et al., 2015). Polarizable MD simulations further improved the accuracy of the underlying force fields, especially for dynamic properties (Schröder and Steinhauser, 2010; Schröder et al., 2011; Bedrov et al., 2019).

One drawback of classical force fields is the fixed topology, which means bonds are not designed to build or break. There are different approaches how to deal with that: Reactive force fields (REAX-FF) (Russo Jr and Van Duin, 2011; Zhang et al., 2014; Weismiller et al., 2015) have been developed, which use bond orders to describe the formation or breaking of bonds. In condensed-phase system, proton transfer has been modeled by applying a Markov model on top of molecular dynamics simulations (Dreßler et al., 2020a; Dreßler et al., 2020b) Alternatively, alchemical mutations with single or dual topology approaches can be applied if topology changes are required (Boresch and Karplus, 1999a; Boresch and Karplus, 1999b; Shirts, 2012; Mey et al., 2020). Alchemical approaches typically use an alchemical coupling parameter *λ* to control the transition of one molecule into another one (including possible bond break/formation); in our case, the transition from the protonated to the deprotonated species or *vice versa*. In constant pH simulations, the (de-)protonation reaction may be described as an instantaneous protonation state change (Mongan et al., 2004) or using alchemical intermediates (Lee et al., 2004; Khandogin and Brooks, 2005; Mongan and Case, 2005; Radak et al., 2017; Dobrev et al., 2020).

However, almost all these approaches are usually applied to a solute with few (de-)protonation sites. Often these sites are coupled to a “proton bath” (Börjesson and Hünenberger, 2001; Donnini et al., 2016; Radak et al., 2017) or an implicit solvent (Mongan et al., 2004; Mongan and Case, 2005) to ensure charge neutrality of the simulation box. However, this coupling limits the number of (de-)protonation sites, which is fine for constant pH simulation of an aqueous protein solution but maybe not be appropriate anymore if all solvent molecules are subject to the proton transfers. This is particularly true for protic ionic liquids (PILs), as proton transfers must be adequately captured even though hundreds of (de-)protonation sites exist. PILs consist of a Brønsted base (B) and acid (HA) and, therefore, can exchange a proton, which is a reaction currently not featured by modern force fields. In general, this reaction reads as

$$\text{HA}+\text{B}⇌{\text{A}}^{-}+{\text{BH}}^{+}$$ (1)

For example, accounting for proton exchange effects will be crucial for an adequate description of the conductivity in PILs. Additionally, examining the moving proton particularly can gain insight into the mechanism. However, classical constant pH simulations cannot model proton hopping from one molecule to another. Multistate empirical valence bond models for water (Schmitt and Voth, 1998; Day et al., 2002) focus on the moving proton and its delocalization between different water molecules. Still, they cannot cope with a large number of different (de-)protonation sites in protic ionic liquids.

We present protex - an open-source Python-based tool for proton exchange in MD simulations. It works seamlessly with the OpenMM toolkit (Eastman et al., 2017) and can perform customized transfer reactions without restricting the number of (de-)protonation sites. Contrary to common Monte Carlo approaches (Baptista et al., 2002; Mongan et al., 2004) of accepting/denying the proton transfers, we perform the one-step proton hopping with a quantum-mechanically derived probability once a distance criterion between the hopping proton and the acceptor is met. However, our probabilities are determined by a Markov chain model [Jacobi et al. (2022)]. In contrast to Dreßler et al, (2020a) and Dreßler et al. (2020b) our Markov chain is applied prior to MD simulation to compute reasonable starting probabilities for the various reactions. Subsequently, the probabilities can be set manually to test several models for proton diffusion and to optimize the agreement with the experiment. The program package protex is freely available on GitHub (https://github.com/cbc-univie/protex).

## 2 Materials and methods

### 2.1 *λ*-states of protic ionic liquids in a single topology approach

**FIGURE 1:**
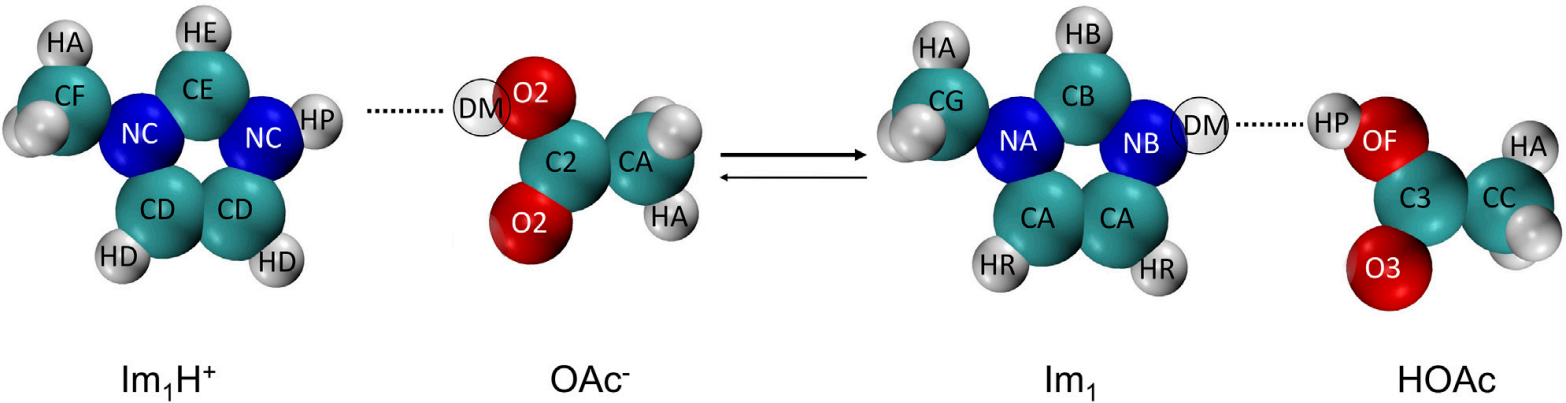
The proton transfer reaction of 1-methylimidazolium (Im_1_H^+^) and acetate (OAc^−^) yielding 1-methylimidazole (Im_1_) and acetic acid (HOAc). For the sake of simplicity, the abbreviated names of the atom types are used (see Table 1).

The protic ionic liquid 1-methylimidazolium acetate [Im_1_H][OAc] is in equilibrium with its neutral species 1-methylimidazole Im_1_ and acetic acid HOAc as shown in Figure 1.

The program protex uses a single topology approach with two discrete *λ*-states to allow for the proton exchange. For imidazoles and acetate, we model the neutral species Im_1_ and HOAc and the cation Im_1_H^+^ and anion OAc^−^, respectively. In principle, it is also possible to model the protonated acetic acid (Ingenmey et al., 2018; Jacobi et al., 2022), which might be necessary for the Grotthus conductivity mechanism, but we restrict ourselves to the simple protonation scheme by Jacobi et al. (2022) for the sake of simplicity. The deprotonation of the Im_1_H^+^ or HOAc is modeled by turning the hydrogen (HP) into a dummy atom (DM) which is part of the acetate OAc^−^ and imidazole Im_1_ molecule.

In contrast to common alchemical mutations for proton transfer, the presented approach is not limited to partial charge mutations (Mey et al., 2020). As the atom types are changed to fit the DGenFF force field nomenclature (Chatterjee et al., 2019; Lin and MacKerell, 2019; Kumar et al., 2020) of the charged/neutral species, all bonded and non-bonded parameters are modified. Tables 1, 2 outline these changes in the atom types, Lennard-Jones parameters, partial charges *q*_*iβ*_, and polarizabilities *α*_*iβ*_. In imidazolium, both ring nitrogens share the same atom type NC. The neutral imidazole Im_1_ has lone pairs at the unsubstituted ring nitrogen (NB). As a consequence of protonation, the charge of these lone pairs is set to 0 e, turning off all their interactions.

**TABLE 1:** Atomtype, corresponding abbreviation, and Lennard-Jones parameters for the molecules Im_1_H^+^ and Im_1_, OAc^−^, HOAc. LP are lone pairs belonging to the respective nitrogens and oxygens.

Im_1_H^+^				Im_1_			
Type	Abbr	*ɛ* [*kcal*/*mol*]	*r*_min_/2 [Å]	Type	Abbr	*ɛ* [*kcal*/*mol*]	*r*_min_/2 [Å]
CD33F	CF	−0.0486	2.040	CD33G	CG	−0.0513	2.040
HDA3A	HA	−0.0240	1.340	HDA3A	HA	−0.0240	1.340
ND2R5C	NC	−0.0791	1.850	ND2R5A	NA	−0.0578	1.861
CD2R5D	CD	−0.0329	1.800	CD2R5A	CR	−0.0523	2.070
HDR5D	HD	−0.0350	0.700	HDR5A	HR	−0.0550	1.250
CD2R5E	CE	−0.0597	1.850	CD2R5B	CB	−0.0680	1.980
HDR5E	HE	−0.1000	0.550	HDR5B	HB	−0.0870	1.103
				ND2R5B	NB	−0.0511	1.956
HDP1A	HP	−0.0100	0.400	DUMH	DM	−0.0000	0.010
LPD	LP	−0.0000	0.010	LPD	LP	−0.0000	0.010

OAc^−^				HOAc			
Type	Abbr	*ɛ* [*kcal*/*mol*]	*r*_min_/2 [Å]	Type	Abbr	*ɛ* [*kcal*/*mol*]	*r*_min_/2 [Å]
CD2O2A	C2	−0.1566	1.800	CD2O3A	C3	−0.0560	1.650
CD33A	CA	−0.0337	2.040	CD33C	CC	−0.0195	1.940
HDA3A	HA	−0.0240	1.340	HDA3A	HA	−0.0240	1.340
OD2C2A	O2	−0.1575	1.910	OD2C3A	O3	−0.1141	1.880
				OD31F	OF	−0.1090	1.710
DUMH	DM	−0.0000	0.010	HDP1A	HP	−0.0100	0.400
LPD	LP	−0.0000	0.010	LPD	LP	−0.0000	0.010

**TABLE 2:** Partial charges, polarizabilities and Thole parameters for the molecules Im_1_H^+^ and Im_1_, OAc^−^, HOAc. LP are lone pairs belonging to the respective nitrogens and oxygens.

Im_1_H^+^					Im_1_				
Atom	Type	*q*_*iβ*_ [*e*]	*α*_*iβ*_ [Å^3^]	Thole [Å]	Atom	Type	*q*_*iβ*_ [*e*]	*α*_*iβ*_ [Å^3^]	Thole [Å]
C1	CD33F	−0.182	−1.181	1.1	C1	CD33G	−0.161	−1.081	1.0
H1	HDA3A	0.135			H1	HDA3A	0.094		
H2	HDA3A	0.135			H2	HDA3A	0.094		
H3	HDA3A	0.135			H3	HDA3A	0.094		
N1	ND2R5C	0.158	−0.803	1.0	N1	ND2R5A	0.140	−1.063	1.3
C2	CD2R5D	−0.107	−1.083	1.1	C2	CD2R5A	−0.369	−1.378	1.3
H4	HDR5D	0.195			H4	HDR5A	0.150		
C3	CD2R5D	−0.047	−1.083	1.1	C3	CD2R5A	0.188	−1.378	1.3
H5	HDR5D	0.192			H5	HDR5A	0.053		
C4	CD2R5E	−0.023	−1.253	1.2	C4	CD2R5B	0.118	−0.868	1.3
H6	HDR5E	0.203			H6	HDR5B	0.073		
N2	ND2R5C	−0.157	−0.803	1.0	N2	ND2R5B	0.000	−0.840	1.0
H7	HDP1A	0.363			H7	DUMH	0.000		
LPN21	LPD	−0.000			LPN21	LPD	−0.474		

OAc^−^					HOAc				
Atom	Type	*q*_*iβ*_ [*e*]	*α*_*iβ*_ [Å^3^]	Thole [Å]	Atom	Type	*q*_*iβ*_ [*e*]	*α*_*iβ*_ [Å^3^]	Thole [Å]
C1	CD2O2A	0.708	−1.016	0.899	C1	CD2O3A	0.858	−1.207	0.708
C2	CD33A	−0.194	−1.681	1.414	C2	CD33C	−0.300	−2.114	0.750
H1	HDA3A	0.004			H1	HDA3A	0.092		
H2	HDA3A	0.004			H2	HDA3A	0.092		
H3	HDA3A	0.004			H3	HDA3A	0.092		
O1	OD2C2A	0.003	−0.699	2.399	O1	OD2C3A	0.000	−0.922	1.539
O2	OD2C2A	0.003	−0.699	2.399	O2	OD31F	0.000	−1.280	1.124
H	DUMH	0.000			H	HDP1A	0.374		
LPO11	LPD	−0.383			LPO11	LPD	−0.319		
LPO12	LPD	−0.383			LPO12	LPD	−0.319		
LPO21	LPD	−0.383			LPO21	LPD	−0.285		
LPO22	LPD	−0.383			LPO22	LPD	−0.285		

All these changes ensure that the molecules behave according to their charge state. This is particularly crucial for ionic liquids as the Coulombic interactions are neither short-ranged nor restricted to ion pairs and lead to cage-like structures (Schröder, 2011; Szabadi et al., 2022).

### 2.2 Polarizable force fields

During the protex update of the *λ*-states, significant changes in the atomic charges occur (see Table 2), which turn molecular ions into neutral molecules and *vice versa*. Since such drastic changes in electrostatic interactions between the molecules destabilize MD simulations, polarizable forces were applied to smoothen the transition of the Coulomb energy. These polarizable forces are anyway essential to close the gap between computational and experimental dynamics as non-polarizable force fields are usually one order of magnitude to viscous (Bedrov et al., 2019).

Among the different approaches to introduce polarizability to an MD simulation, we used the Drude oscillator model (Lamoureux and Roux, 2003; Bedrov et al., 2019). Each polarizable atom *iβ* is assigned an additional pair of Drude particles evoking an induced dipole ${\vec{\mu }}_{i\beta }^{\mathrm{ind}}$ . One Drude particle is located at the site of the atom itself with a charge of −*q* ^*δ*^. The second Drude particle carries the opposite charge + *q* ^*δ*^, which is generally negative as *q* ^*δ*^ < 0e. The second Drude particle is attached by a harmonic spring to the first. The corresponding force constant $k_{i\beta }^{\delta }$ of the spring is given by

$$k_{i\beta }^{\delta }=\frac{1}{4\pi \epsilon_{0}}\frac{{\left(q^{\delta }\right)}^{2}}{\alpha_{i\beta }}$$ (2)

**FIGURE 2:**
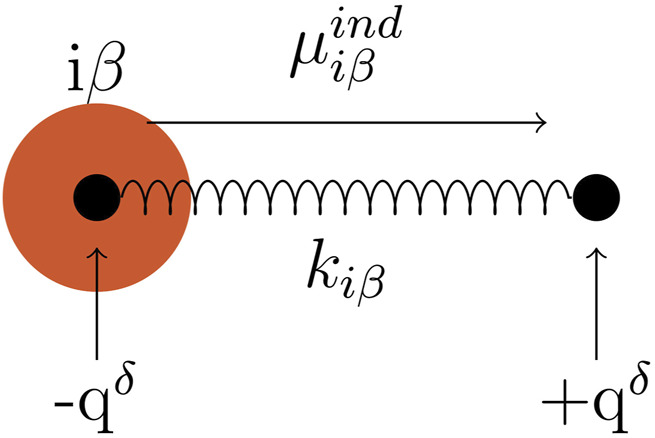
Drude pair, represented by black filled circles, connected with a harmonic spring with the spring constant k_*iβ*_. One Drude particle is located at the atom i*β* (represented as an orange circle), carrying the charge -q^*δ*^. The other Drude particle carries the charge +q^*δ*^. The induced dipole ${\vec{\mu }}_{i\beta }^{\mathrm{ind}}$ points from the Drude particle at the atom site to the mobile one, as indicated by the arrow.

And usually set to a constant value for all atoms in a simulation resulting in increasing Drude charges *q* ^*δ*^ with increasing polarizabilities *α*_*iβ*_. The induced dipole ${\vec{\mu }}_{i\beta }^{ind}$ depends on the charge *q* ^*δ*^ and the distance ${\vec{d}}_{i\beta }$ between the Drude particles: ${\vec{\mu }}_{i\beta }^{\mathrm{ind}}=q^{\delta }{\vec{d}}_{i\beta }$ . It points from the Drude particle located at the polarizable atom to the mobile Drude particle, which can also be seen in Figure 2. The displacement $\left|{\vec{d}}_{i\beta }\right|$ should be much smaller than bond distances and is usually less than 0.1 Å. The total self-polarization energy reads $U^{\delta \delta }=\sum_{i\beta }k_{i\beta }^{\delta }{\left({\vec{d}}_{i\beta }\right)}^{2}$ .

As the Coulomb interaction of the Drude particles already contains the dispersion between molecules, the corresponding Lennard-Jones interactions have to be reduced to counteract double counting. Bypassing a complete reparametrization of the Lennard-Jones parameters, atomic $\epsilon_{i\beta }^{LJ}$ -parameters can be scaled systematically as a function of the polarizability (Becker et al., 2016; Bedrov et al., 2019; Szabadi and Schröder, 2021; Joerg and Schröder, 2022):

$$\epsilon_{i\beta }^{LJ}=\epsilon_{i\beta }^{LJ,nonpol}\frac{\Delta \alpha +s\;\alpha_{\max}}{s\;\Delta \alpha +\alpha_{\max}}$$ (3)

Using the largest atomic polarizability *α*_max_ and the difference Δ*α* between *α*_max_ and the polarizability *α*_*iβ*_ of the current polarizable atom. The scaling factor *s* determines the influence of the polarizability on the Lennard-Jones $\epsilon_{i\beta }^{LJ}$ (Becker et al., 2016; Bedrov et al., 2019; Szabadi and Schröder, 2021; Joerg and Schröder, 2022).

### 2.3 The program package protex

**FIGURE 3:**
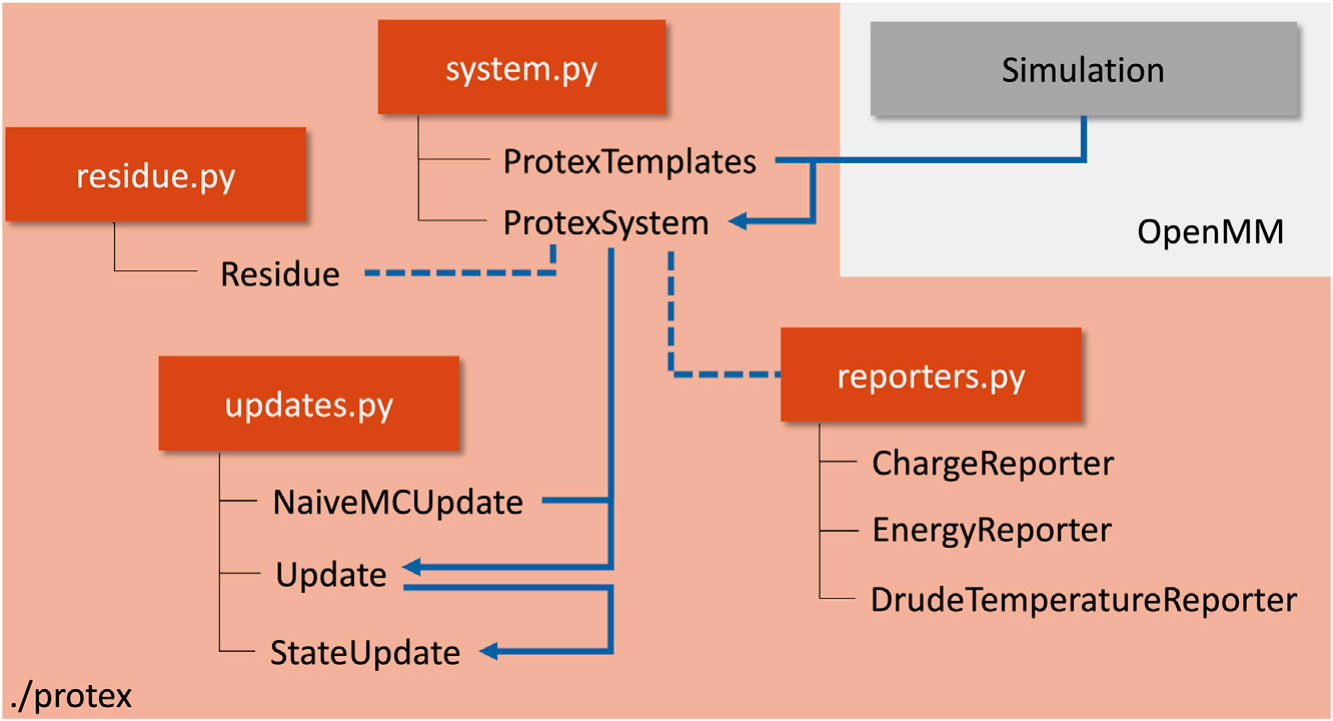
Flowchart of a typical setup to simulate a protic solvent using protex. Python modules are shown in orange, with the corresponding classes denoted below. The blue arrows indicate a typical workflow to run a protex simulation. The gray box visualizes the external simulation object, which is not part of protex itself. A concrete example can be found in the SI.

Protex augments an OpenMM simulation object and is not restricted to simulations of ionic liquids. The two main parts of the program are the ProtexSystem and Update classes. The former gathers the simulation object and additional information on the update process, wrapped in the ProtexTemplates class. The latter is responsible for the actual update process and handles the logic during an update. Figure 3 gives an overview of the program package protex.

The system object was created using CHARMM topology and parameter files in this work. A condition to perform proton exchange between residue protonation states is that the residues prone to a proton exchange have a one-to-one mapping between their atoms in the protonated and deprotonated form of the topology file. Please find a detailed example in the documentation at GitHub (https://github.com/cbc-univie/protex).

The ProtexTemplates class is used to gather the additional information needed for the simulation. The user may specify which transfer reactions should occur by specifying the residue names, the maximum distance, and the probability of this reaction. This way, the back-and-forth reaction of, for example, Im_1_H^+^ + OAc^−^ → Im_1_ + HOAc, can be defined independently of the reaction Im_1_ + HOAc → Im_1_H^+^ + OAc^−^. Additionally, the atom name of the donor/acceptor atom needs to be specified. This would be the hydrogen for Im_1_H^+^ and the nitrogen for Im_1_ or the hydrogen of the acetic acid and both oxygens of the acetate. An example for the concrete definition of these variables can be found in the SI.

The ProtexSystem class combines the two former objects. It serves as an anchor for the actual propagation of the simulation, stores all information on the individual molecules (*e.g.*, current name, charges, parameters, … ) in a separate Residue class, and can be used for loading and saving the current state and a PSF file. Two additional reporters are available, one reporting the current charge of all molecules in the system (ChargeReporter) and one reporting the energy contributions of the individual force objects (EnergyReporter). They can be used similarly to any other OpenMM reporter.

The Update classes handle everything connected to the update process during the simulation. The abstract base class Update serves as an anchor for different concrete implementations. NaiveMCUpate was used in this study, which checks for updates based on the distance and probability criterion. If the distance between the acceptor and donor falls below the distance criterion (as defined in ProtexTemplates), the proton exchange will happen with the given probability. The StateUpdate is responsible for the actual updates. It can be called anytime during the propagation of the trajectory. The update can either happen instantaneously between protonation states or using a non-equilibrium protocol in which multiple intermediate *λ*-states are used to interpolate between a source and target protonation state smoothly. The user can specify if only partial charges or all non-bonded and bonded interactions should be changed between protonation states.

As found in our previous study, the equilibrium for the Im_1_H^+^/OAc^−^ system is around 30% charged and 70% neutral species. Hence an optional mechanism to stay around this equilibrium was implemented. As reported by Lill and Helms (Lill and Helms, 2001), the energy barrier for (de-)protonation is a function of the local environment and is not restricted to the exchanging molecules. Strictly speaking, the position of the barrier maximum is also a function of the local environment (Lill and Helms, 2001). However, as the corresponding calculations result in significant computational effort, we start with a fixed distance criterion. Dreßler et al, (2020a) and Dreßler et al. (2020b) introduced a Fermi function based to model the probability as a function of the distance, which will be included in future versions of protex.

**FIGURE 4:**
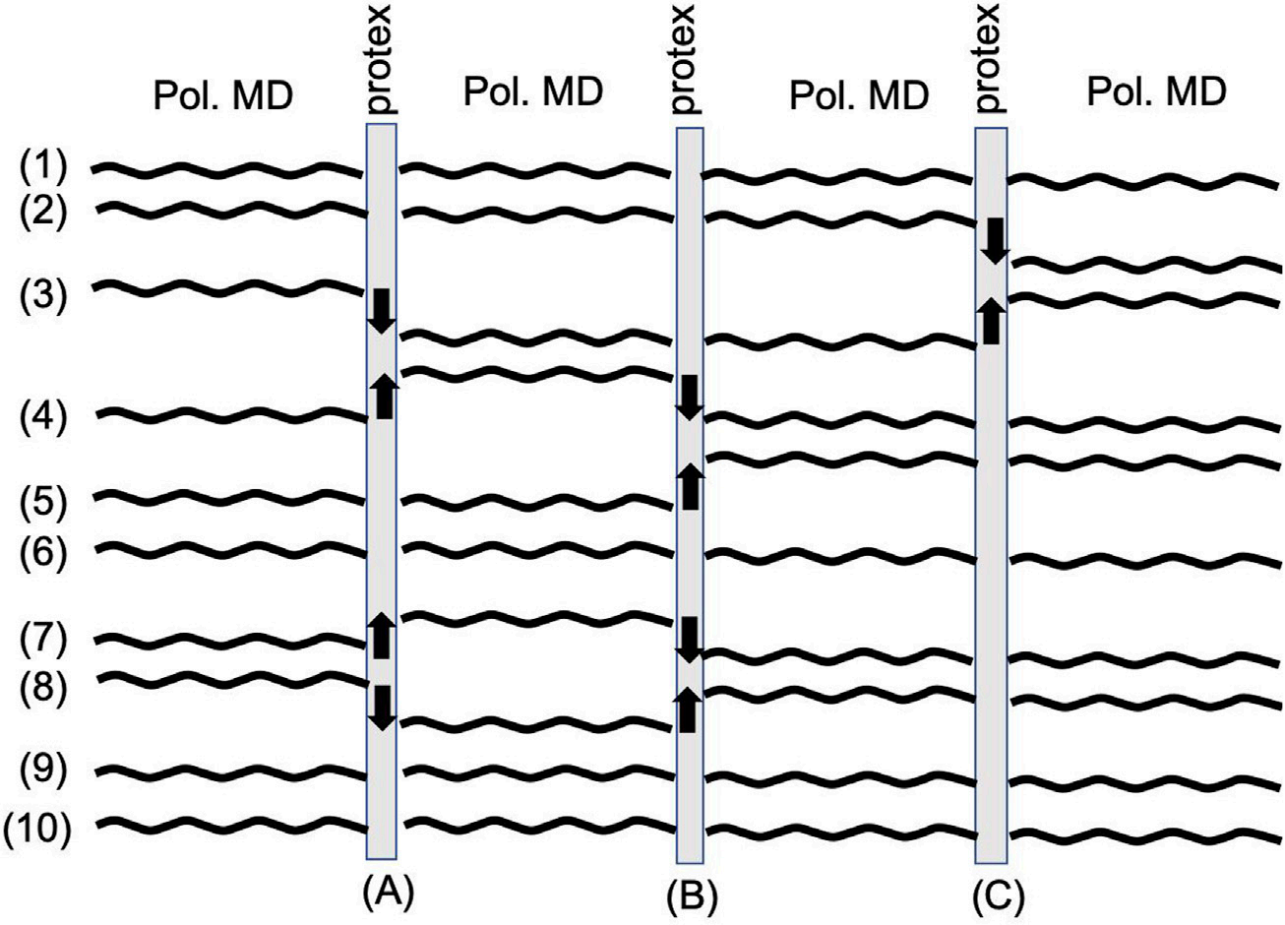
Workflow of a typical protex simulation. A classical polarizable MD simulation in OpenMM is stopped at regular time intervals. protex determines possible molecules for proton transfers. An up arrow depicts one of the protonations, and a down arrow one of the deprotonations. The force field parameters are changed to represent the new (de-)protonated species, and the classical polarizable MD simulation is continued until the next proton transfer event.

The current probability *p*_*ref*_ is updated at each proton exchange event (see Figure 4)

$$p=p_{\mathrm{ref}}+c\cdot {\left(\frac{n_{k}^{\mathrm{now}}}{n_{k}^{\mathrm{ref}}}-1\right)}^{3}$$ (4)

where $n_{k}^{\mathrm{now}}$ and $n_{k}^{\mathrm{ref}}$ are the current and reference (initial) number of molecules of species *k* and *c* is a tunable prefactor. The power of three ensures the sign stays the same and allows for increased or decreased probabilities *p*: A ratio $n_{k}^{\mathrm{now}}/n_{k}^{\mathrm{ref}}$ below unity indicates that the number of the corresponding species *k* is below average. Hence, a reaction of that species should occur less often, which is realized by the reduced probability of this reaction due to the negative bracket in Eq. 4. On the other hand, more molecules than the reference indicate too few reactions. Hence the positive factor increases the probability of the reaction. Protex is designed to model proton transfers in a solvent at room temperature. Quantum effects at lower temperatures may only be indirectly modeled by changing the distance criterion and probability for particular reactions.

Figure 4 shows the typical workflow of a protex simulation. Each number depicts the trajectory of one species in the system. After some specified simulation time (A), protex checks for possible proton transfers and executes them (indicated by the black arrows in Figure 4). Then the simulation is propagated until the next update event (B). Here, some of the molecules may have stayed close to each other and exchanged the proton back (see trajectory (7) and (8) in Figure 4). However, it is also possible that the proton is transferred to the next molecule [see trajectory (3)–(4)–(5)]. A significant amount of molecules never face a proton exchange [see trajectory (1), (6), (9), and (10)] which may be due to unfavorable orientations or no corresponding partner. The number of protonations equals the number of deprotonations, as the overall system is neutral at all times. Consequently, the number of up arrows is the same as that of down arrows in Figure 4. Also, the total number of protonations/deprotonations may differ between two exchange events. For example, (C) in Figure 4 has fewer exchanges than (A) or (B).

Benchmark tests on a NVIDIA RTX3090 and AMD Threadripper with a typical setup of 10 ps simulation time between the updates, showed that the protex routine takes about 25% of the total simulation time. Details can be found in the SI.

### 2.4 Computational setup

Details on the parametrization process of the molecular species involved can be found in Joerg and Schröder (2022). In short, the force field for Im_1_H^+^OAc^−^ was based on the CHARMM General Force Field (CGenFF) (Kumar et al., 2020). Since the ionic liquid is not fully featured in the standard force field, electrostatic and bonded parameters were optimized based on quantum-mechanical reference calculations. For the calculation of dynamics properties, polarizable MD simulations were utilized. The polarizability was implemented using the Drude model, which adds an additional harmonic spring to all non-hydrogen atoms to emulate the induced forces. Due to their low mass, hydrogen atoms cannot be made polarizable, so the respective polarizabilities are added to their corresponding parent atoms. Drude particles were assigned a mass of 0.4 μ and a force constant $k_{i\beta }^{\delta }$ = 1,000 kcal/mol/Å^2^, (squared Angstrom). For stability reasons, the maximum distance for the mobile Drudes was set to 0.25 Å. Lennard-Jones interactions were reduced as described in Joerg and Schröder (2022), using Eq. 3. Scaling factors *s* of 0.25 and 0.4 were employed, each with five replicas and a simulation time of 50 ns Each system contained 1,000 molecules, resulting in 150 Im_1_H^+^/OAc^−^ and 350 Im_1_/HOAc each (representing the initial 30%:70% equilibrium) as shown in Table 3.

**TABLE 3:** Systems under investigation. All systems contain a total of 1,000 molecules, with initially 30% Im_1_H^+^ and OAc^−^, and 70% Im_1_ and HOAc. Scaling factors *s* of 0.25 and 0.4 were used, with five replicas and 50 ns simulation time each.

*s*	Initial Im_1_H/OAc^−^	Initial Im_1_/HOAc		Sim. Period
	[# molecules]		[# replica]	[ns]
0.25	150	350	5	50
0.40	150	350	5	50

Packmol (Martínez et al., 2009) was used to pack the initial simulation boxes, which were subsequently subject to energy minimizations using CHARMM, removing possible clashes or very unfavorable configurations of molecules (Brooks et al., 2009). Then, the polarizable system was equilibrated with OpenMM for 5 ns applying a Monte-Carlo barostat at 1.0 atm to determine the final box length. The production runs in the NVT ensemble were done in OpenMM with a time step of 0.5 fs for 50 ns Temperature control of polarizable systems with the conventional Dual-Nosé-Hoover thermostat (Martyna et al., 1992) is challenging, due to heat flow from the degrees of freedom of real atoms to Drude atoms. This causes the center-of-mass temperature to be overestimated. Hence, we used a temperature-grouped Dual-Nosé-Hoover thermostat as described by Son et al. (2019) and Gong and Padua, (2021), which adds an additional group for center-of-mass translations, thus improving the accuracy of the simulations. The temperature was set to 300 K for the real atoms and 1 K for the Drude particles. Electrostatic interactions were treated using the Particle Mesh Ewald method: The cut-off distance was set to 11 Å and the switch distance to 10 Å. All simulations were run on the CUDA platform in single precision. Further details on the setup can be found in Joerg and Schröder, (2022).

Four possible transfer reactions were defined, including the forward and backward reaction described by Eq. 1 as well as the transfer between Im_1_H^+^/Im_1_ and HOAc/OAc^−^. In this work, the protonation states were switched instantaneously, with no additional *λ* states between the initial and final state. In the first step at each transfer event (see Figure 4), distances between transferable hydrogen atoms and hydrogen acceptors (nitrogen/oxygen) of the other molecules were checked, and only those pairs with a distance lower than 1.55 Å considered for the next step. The second step involves proton transfers with a particular probability. The initial probability of Table 4 are in accordance with Jacobi et al. (2022) but are updated applying Eq. 4. The time interval between the transfer checks was set to 10 ps

**TABLE 4:** We consider four possible proton transfer reactions corresponding to the simple reaction scheme in Jacobi et al. (2022). According to Eq. 4, the probabilities are updated during simulations. The first two reactions change the number of charged molecules.

Reactants	Products	*r*_max_ [Å]	Probability *p*_*ref*_ (%)	*c*
Im_1_H^+^ + OAc^−^	Im_1_ + HOAc	1.55	99.4	300
Im_1_ + HOAc	Im_1_H^+^ + OAc^−^	1.55	9.8	300
Im_1_H^+^+Im_1_	Im_1_+Im_1_H^+^	1.55	20.1	300
HOAc + OAc^−^	OAc^−^ + HOAc	1.55	68.4	300

### 2.5 Analysis

For the analysis of the trajectories, the MDAnalysis package (Michaud-Agrawal et al., 2011; Gowers et al., 2016) was applied and augmented by self-written Python code. For example, the combination of MDAnalysis and the voro++ library (Rycroft, 2009) allows for the computation of the coordination number *N*_*kl*_ and the volume *V*_*k*_(*shell* = 1) of the first solvation shell around molecules of species *k* (Zeindlhofer et al., 2018; Szabadi et al., 2022). Based on this information, a shell-based potential of mean force

$$PMF_{kl}\left(shell=1\right)=-k_{B}T\;\ln\left[\frac{c_{l}\left(shell=1\right)}{c_{l}}\right]$$ (5)

can be computed from the concentration *c*_*l*_ (*shell* = 1) = *N*_*kl*_/*V*_*k*_(*shell* = 1) of species *l* in the first shell around species *k* and the bulk concentration *c*_*l*_ = *N*_*l*_/*V*. Negative *PMF*-values indicate preferential coordination of the species *l* around species *k*, whereas positive values result from a depletion of species *l* around *k*.

The diffusion coefficient can be calculated using the Einstein relation (Allen and Tildesley, 1986). For species *k*, it reads

$$D_{k}=\frac{1}{6}\frac{d}{dt}{\left\langle \Delta r^{2}\left(t\right)\right\rangle }_{k}$$ (6)

With Δ *r*(*t*) = |*r*(*t*) − *r* (0)|. To obtain diffusion coefficients for each species in the system, the possible proton transfers, which consequently change the residue names, had to be accounted for. Therefore, the time series for each residue was cut when a transfer occurred. Only time series with at least 25 ns length were analyzed for the final analysis. Although this reduces the statistics of the mean-squared displacement, it ensures that the mobility of the charged and neutral compounds is not mixed. The slope for Eq. 6 was taken between 2 and 6 ns Additionally, diffusion coefficients for combined Im_1_H^+^/Im_1_ as well as OAc^−^/HOAc were calculated.

**FIGURE 5:**
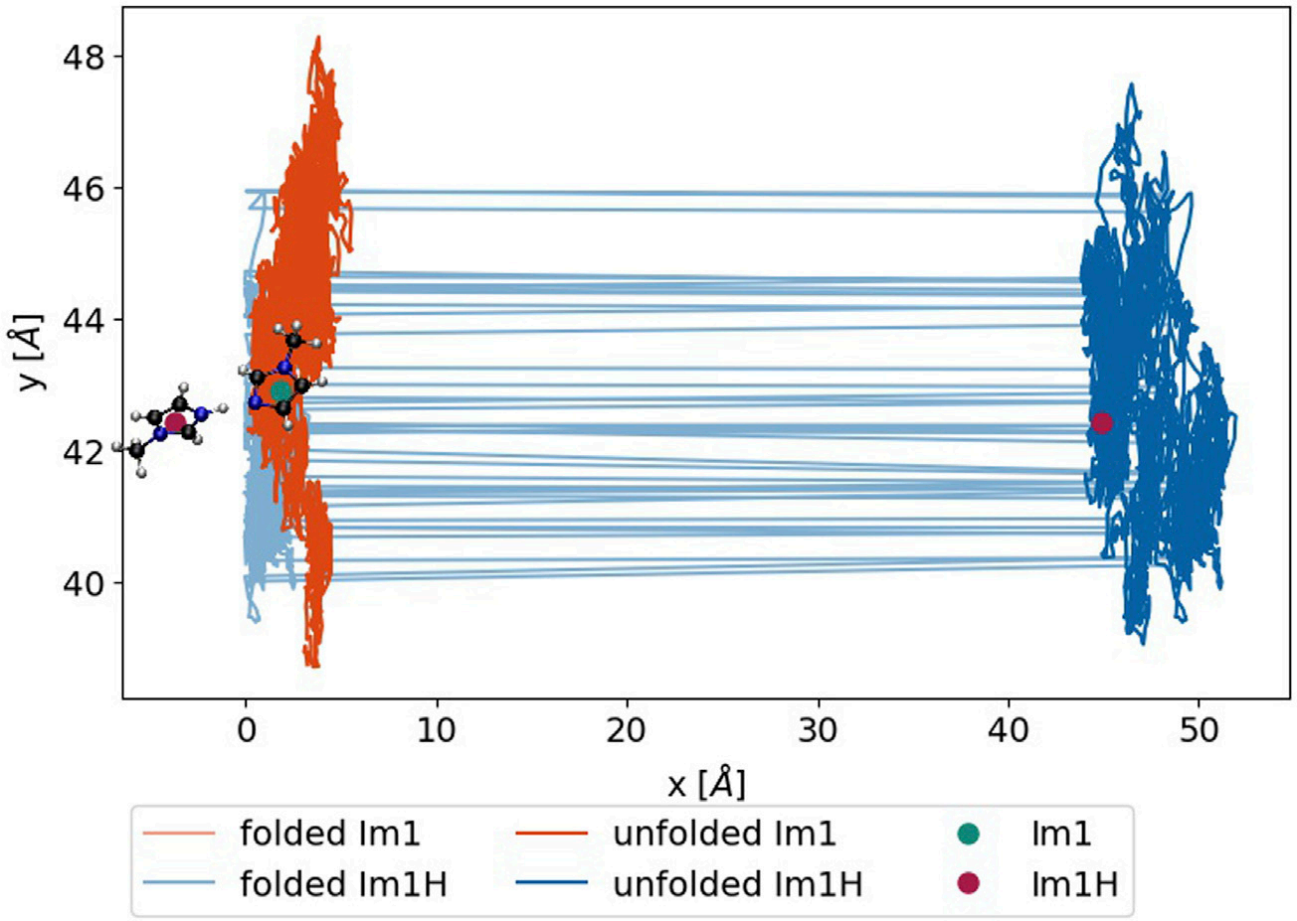
Trajectory of an exemplary Im_1_H^+^ (blue) and Im_1_ (green), either during the simulation (light) or after unfolding the box (dark). The green and red dots denote the position at the time of the update and after the unfolding, respectively.

The analysis of the conductivity *σ*(0) needed some extra attention. Commonly, *σ*(0) is obtained from the mean-squared displacement

$$\sigma \left(0\right)=\frac{1}{6Vk_{B}T}\frac{d}{dt}\left\langle \Delta {\vec{M}}_{J}{\left(t\right)}^{2}\right\rangle$$ (7)

of the collective translational dipole moment ${\vec{M}}_{J}=\sum_{i}q_{i}\;{\vec{r}}_{i}$ using the molecular charges *q*_*i*_ and the respective center-of-masses ${\vec{r}}_{i}$ from the unfolded trajectory. However, the occurrence of proton transfer reactions is decided on the minimum distance using the periodic boundary conditions during the production of the folded trajectory. In Figure 5, a proton transfer between 1-methylimidazole (turquoise dot) and 1-methylimidazolium (red dot) is sketched. The distance between the nitrogen of the Im_1_ and the hydrogen of Im_1_H^+^ is below 1.55 Å considering the periodic boundary conditions. If a proton transfer occurs, the imidazole charge is set to +1e, and the imidazolium becomes neutral. However, after unfolding the trajectory, the distance between the two exchange partners is much larger, and huge jumps in the collective dipole moment occur.

The simplest way to bypass this problem is to undo this huge jump in $\Delta M_{J}^{2}$ of the unfolded trajectory and subsequently add the contributions emerging from the occurred proton transfers. This way, one does not have to bookmark all toroidal jumps. The contribution $\delta {\vec{M}}_{J}$ of each proton transfer is

$$\delta {\vec{M}}_{J}={\vec{M}}_{J}^{a}-{\vec{M}}_{J}^{b}=\left(q_{i}^{a}\cdot {\vec{r}}_{i}^{\;a}+q_{j}^{a}\cdot {\vec{r}}_{j}^{\;a}\right)-\left(q_{i}^{b}\cdot {\vec{r}}_{i}^{\;b}+q_{j}^{b}\cdot {\vec{r}}_{j}^{\;b}\right)$$ (8)

Using the center-of-masses ${\vec{r}}_{i}$ and ${\vec{r}}_{j}$ of the molecules *i* and *j*. The indices *b* and *a* denote before (b) and after (a) the proton transfer. Based on our simple reaction scheme in Table 4 eight different types of these contributions exist, which are tabulated in Table 5.

**TABLE 5:** Correction for the total translational dipole moment. $M_{J}^{b}$ and $M_{J}^{a}$ are the total translational dipole moment before and after the proton transfer, $\delta M_{J}=M_{J}^{a}-M_{J}^{b}$ . Before and After denotes the current total charge of molecules *i* and *j*.

Before		After		$M_{J}^{b}$	$M_{J}^{a}$	*δM*_*J*_
$q_{i}^{b}$ /e	$q_{j}^{b}$ /e	$q_{i}^{a}$ /e	$q_{j}^{a}$ /e			
0	0	+1	−1	0	$-e\cdot {\vec{r}}_{ij}$	$-e\cdot {\vec{r}}_{ij}$
0	0	−1	+1	0	$e\cdot {\vec{r}}_{ij}$	$e\cdot {\vec{r}}_{ij}$
+1	−1	0	0	$-e\cdot {\vec{r}}_{ij}$	0	$e\cdot {\vec{r}}_{ij}$
−1	+1	0	0	$e\cdot {\vec{r}}_{ij}$	0	$-e\cdot {\vec{r}}_{ij}$
−1	0	0	−1	$-e\cdot {\vec{r}}_{i}$	$-e\cdot {\vec{r}}_{j}$	$-e\cdot {\vec{r}}_{ij}$
0	−1	−1	0	$-e\cdot {\vec{r}}_{j}$	$-e\cdot {\vec{r}}_{i}$	$e\cdot {\vec{r}}_{ij}$
+1	0	0	+1	$e\cdot {\vec{r}}_{i}$	$e\cdot {\vec{r}}_{j}$	$e\cdot {\vec{r}}_{ij}$
0	+1	+1	0	$e\cdot {\vec{r}}_{j}$	$e\cdot {\vec{r}}_{i}$	$-e\cdot {\vec{r}}_{ij}$

## 3 Results and discussion

**FIGURE 6:**
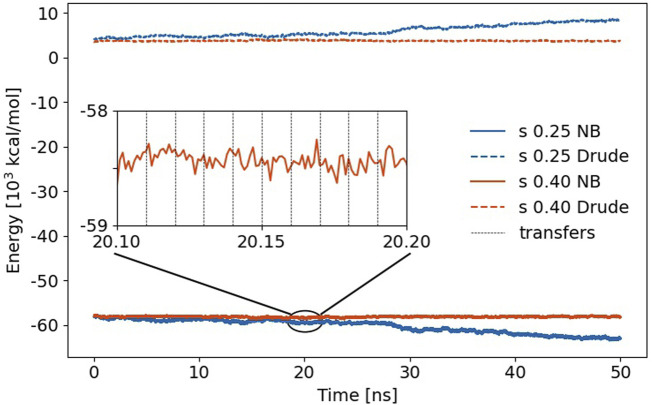
Non-bonded (NB) energy of the trajectories using *s* = 0.25 and 0.4, respectively. The jumps in Coulomb energy at the proton transfer events (black vertical dashed lines) are less than the fluctuations between two transfer events. The dashed lines above 0 kcal/mol represent the self-polarization energy *U* ^*δδ*^.

In MD simulations, proton transfer events are usually harmful non-equilibrium situations as ions may become neutral or *vice versa*. Consequently, one expects significant jumps in the Coulomb energy of the system. This is undoubtedly true for non-polarizable MD simulations, but fortunately, the induced dipoles in polarizable trajectories counteract these jumps and smoothen the non-bonded (NB) interactions as shown in Figure 6 for *s* = 0.25 (blue) and *s* = 0.40 (orange). Strictly speaking, the non-bonded energy also comprises the Lennard-Jones interactions, but these do not change very much during the proton transfer as hydrogens usually have no significant contributions.

The time evolution of this non-bonded energy is a constant profile for *s* = 0.4 and gets more negative for *s* = 0.25. Interestingly, the Drude self-polarization energy, on the other hand, rises about the same amount in that case. Weakening of the Lennard-Jones spheres allows for closer distances of the induced dipoles of two polarizable atoms. Consequently, the interaction of these induced dipoles with other induced dipoles and with the permanent charges results in lower energy. Since this also leads to larger distances between the mobile Drude particle and the polarizable atom, the corresponding self-polarization term *U* ^*δδ*^ increases. These effects in the non-bonded energy and self-polarization cancel out in the total energy, which changes roughly −7 kcal/mol/ns of the complete simulation box in case of *s* = 0.25 which might still be acceptable, although it is almost twice the drift per Drude oscillator compared to water [Lamoureux and Roux (2003)]. However, a scaling factor *s* of 0.40 is preferable as no drift is observed in Figure 6. Zooming into the trajectory (see inset in Figure 6), one notices that the jumps due to the multiple proton exchanges are less compared to the fluctuations of the non-bonded energy between two proton exchange events. This clearly demonstrates the induced dipoles’ functionality for stabilizing proton transfer MD simulations.

**FIGURE 7:**
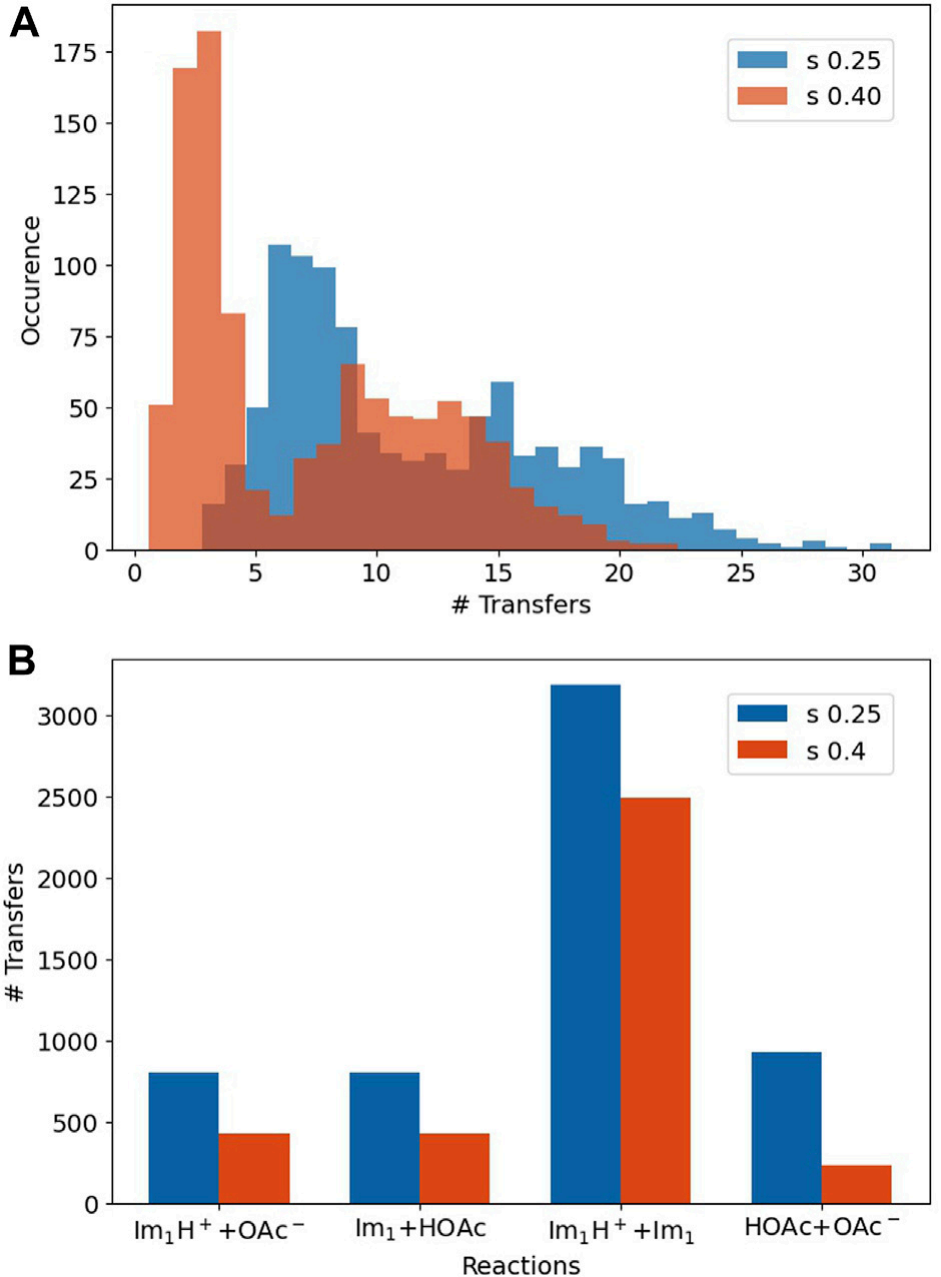
**(A)** The number of transfers for every molecule. **(B)** The number of transfers for the reactions listed in Table 4.

The simulation period of our polarizable MD simulations is 50 ns. As we stop the production every 10 ps to check for proton exchanges, (Jacobi et al., 2022), a molecule may face 5,000 exchanges at maximum. However, the average number of proton transfers for each molecule is much lower (see in the top panel of Figure 7) and equals roughly 10 to 15 exchanges on average.

Please note that the histograms are quite broad, revealing a heterogeneous system. Stronger Lennard-Jones-*s*-scaling leads to more transfers in general for all 500 Im_1_/Im_1_H^+^ and 500 HOAc/OAc^−^ [*s* = 0.25 (blue): 5,725 transfers; *s* = 0.4 (orange): 3,585 transfers] since the overall movement is increased.

The lower panel of Figure 7 depicts the relevance of the reactions in Table 4. Interestingly, the proton transfers are dominated by proton exchanges between imidazole and imidazolium, although the reaction probability is significantly lower than that of the reactions Im_1_H^+^ + OAc^−^ or OAc^−^ + HOAc. This is due to the crucial distance between the hydrogen donor and acceptor, which was set to 1.55 Å in our simulations. As imidazole and imidazolium seem to come closer to each other and have the correct mutual orientation, these reactions happen more often than those with higher probability.

**FIGURE 8:**
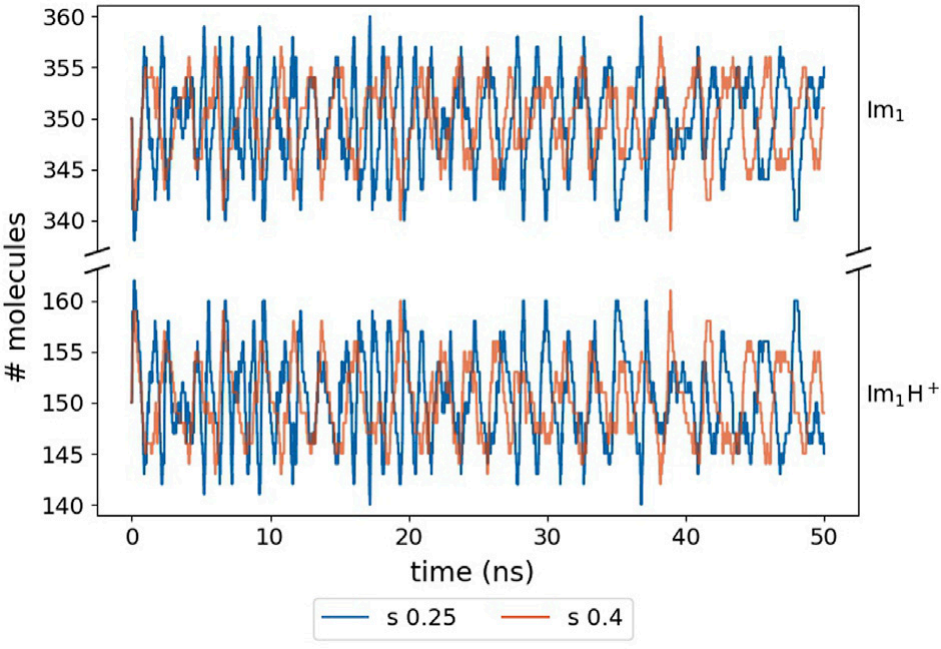
Fluctuating number of Im_1_H^+^ and Im_1_ molecules. The numbers are close to the initial 30% ionic to 70% neutral ratio due to the correction in Eq. 4.

Our simulation still reproduces the equilibrium value of 30% ionic:70% neutral molecules (Jacobi et al., 2022; Joerg and Schröder, 2022) for both *s*-values as shown in Figure 8.

However, we had to apply Eq. 4 to prohibit drifting away from the equilibrium partitioning of the molecules as the total number of proton transfer events, i.e. 5,000, is much lower than in the Markov chain analysis reported by Jacobi et al. (2022). Furthermore, due to the distance criterion *r*_max_ and the mutual orientation of the reacting species in the liquid phase, particular reactions are favored regardless of the value of the reaction probability *p*_*ref*_.

So far, we have shown that our polarizable MD simulations, including proton transfer, are stable for at least 50 ns with the correct ratio of charged and neutral molecules. However, the more interesting question is: What is the difference between a polarizable simulation with fixed molecular charges *q*_*i*_ and our new simulations, including proton transfers?

Table 6 shows the box length *L*, density *ρ* and conductivity *σ*(0) of the systems for scaling factors of *s* = 0.25 and *s* = 0.4. The box length and, thus, density are very similar for the different replicas, as well as compared to the simulations without proton transfer in our previous study (Joerg and Schröder, 2022). This is expected since the same workflow was used, and no proton transfers were allowed during the NpT runs, opposite to the NVT production run, which was used for analyzing transport properties. Hence, the conductivity is expected to differ. A notable increase was found for both systems allowing proton transfers as displayed in Table 6. Since conductivity is a collective property, the statistics are challenging explaining the slight deviations for the different replicas. Interestingly, the standard deviations in the case of *s* = 0.25 are significantly larger. Also, the conductivity *σ*(0) for *s* = 0.25 is above the experimental value, whereas *σ*(0) for *s* = 0.4 is within the range of the measured values.

**TABLE 6:** Average box length *L*, density *ρ* and conductivity *σ*(0) of the different replica for both scaling factors. The reference values for the polarizable MD simulation without proton transfers are taken from Joerg and Schröder (2022). The experimental density is from Chen et al. (2014) and the conductivity from MacFarlane et al. (2006); Hou et al. (2011); Chen et al. (2014); Thawarkar et al. (2019).

	*s* = 0.25			*s* = 0.4		
Rep	*L*	*ρ*	*σ*(0)	*L*	*Ρ*	*σ*(0)
	[Å]	[g cm^−3^]	[mS cm^−1^]	[Å]	[g cm^−3^]	[mS cm^−1^]
Joerg and Schröder, (2022)	48.59	1.03	2.9	48.29	1.05	2.4
1	48.53	1.04	4.3	48.29	1.05	3.2
2	48.59	1.03	6.9	48.30	1.05	4.0
3	48.56	1.04	6.3	48.34	1.05	3.4
4	48.56	1.04	6.5	48.32	1.05	3.3
5	48.56	1.04	5.2	48.31	1.05	3.8
Avg	48.56	1.04	5.8 ± 1.1	48.31	1.05	3.6 ± 0.4
Exp		1.07	3.3–4.4		1.07	3.3–4.4

**FIGURE 9:**
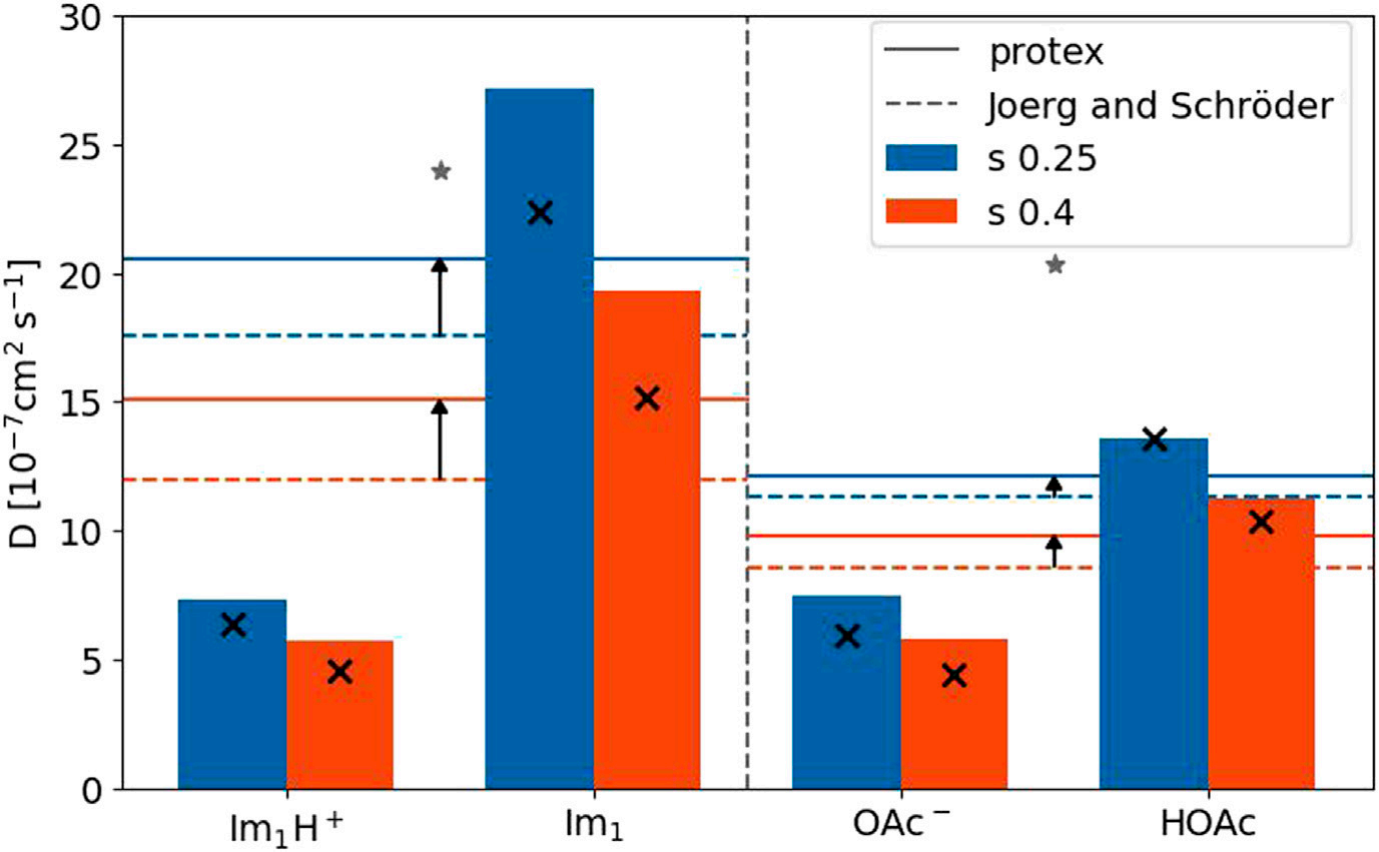
Diffusion coefficients for the four species with a scaling factor of 0.25 and 0.4. The reference values for the single species (black x) are taken from Joerg and Schröder (2022). The experimental values (gray stars) are taken from Thawarkar et al. (2019).

Figure 9 depicts the diffusion coefficients of the four involved species for both *s*-scaling factors. The horizontal solid lines are the average diffusion coefficient of the imidazole-based and carboxylate-based molecules taking into account the different mole fractions and proton transfers. The dashed lines correspond to the polarizable simulations without proton transfers (Joerg and Schröder, 2022). Although the diffusion coefficients increased compared to Joerg and Schröder (2022) (black arrows), they are still smaller than the corresponding experimental value (gray star) for both species (Thawarkar et al., 2019). As expected, the diffusion coefficients of the neutral molecules are higher than their charged counterpart because of fewer Coulombic interactions. This was also true for the polarizable simulations without proton transfers (black x in Figure 9) (Joerg and Schröder, 2022). Except for imidazole, the diffusion coefficients of the species are more or less unaffected by the implementation of the proton transfers. Overall, the molecular translational motion characterized by the diffusion coefficients is not responsible for the increase in the conductivity, which has to be due to collective effects.

**FIGURE 10:**
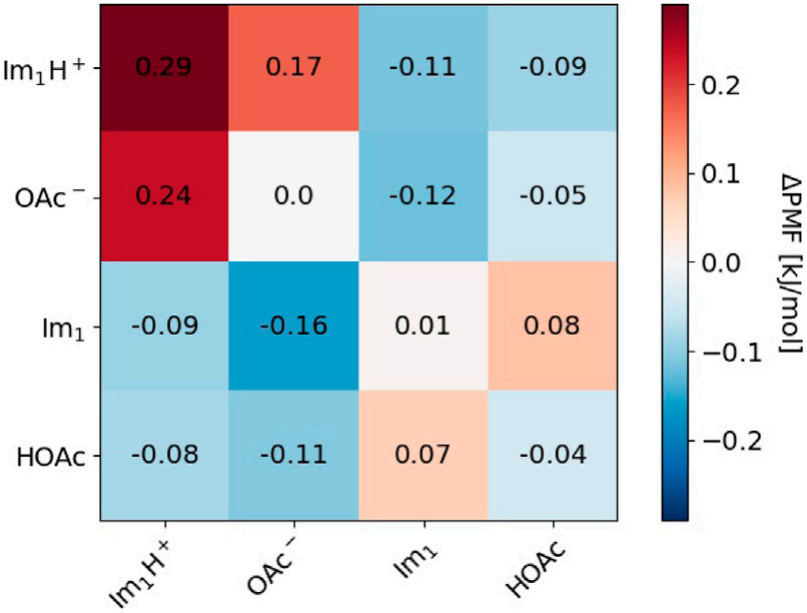
Difference in Potential of mean force (PMF) for *s* = 0.25 between this work and Joerg and Schröder (2022).

Cage effects can be characterized by the shell-resolved potential of mean force *PMF*. Since we are interested in the different behavior of polarizable simulations with and without proton transfer, we plotted the differences Δ*PMF* of the mutual shell-resolved potential of mean forces *PMF*_*kl*_ (*shell* = 1) for the species *k*, *l* ∈ {Im_1_H^+^, OAc^−^, Im_1_, HOAc} as a heat map in Figure 10.

Red boxes indicate that the solvation became less favorable after including proton transfers, whereas blue boxes reveal an increased attraction compared to the simulations without proton transfer. For the conductivity, the Δ*PMF*s, including the charged species in the top left regions of the heat map, are the most interesting. The dark red boxes for Im_1_H^+^/OAc^−^ and Im_1_H^+^/Im_1_H^+^ correspond to weaker coordination of these species and fewer cation/anion or cation/cation pairs increase the conductivity *σ*(0). The overall charge of cation/anion pairs is zero; consequently, this pair does not contribute to *σ*(0). If two cations stick together for a long time, their overall mobility is reduced, hence the electric current. Allowing for proton transfer in the polarizable simulations has multiple effects: First, Im_1_H^+^/OAc^−^ may react and become two neutral molecules. This reaction does not increase the conductivity. Second, in the case of a cation/cation pair, one of the imidazoliums may exchange its proton with acetate. Now, the second imidazolium has a new imidazole and acetic acid neighbor and may be more mobile than in the cation/cation aggregate before. This would increase the conductivity. Quite generally, in Figure 10, neutral molecules seem to accumulate in the immediate neighborhood of charged molecules (blue boxes in the top right region of the heat map) as a consequence of the multiple proton transfer events. This fact shows the weakening of ion cages by proton transfer reactions.

The proton transfers promote the diffusion of imidazoles Im_1_. If an imidazolium inside an ion cage transfers its acidic proton to one of the acetates, the emerging Im_1_ still encounters many other acetates in the former ion cage. This situation is energetically unfavorable, and the imidazole will try to escape immediately, thereby increasing the diffusion coefficient. The corresponding Δ*PMF* is −0.16 kJ·mol^−1^ (see Figure 10).

## 4 Conclusion

The lightweight open-source Python package protex was successfully implemented for polarizable MD simulations of the protic ionic liquid 1-methylimidazolium acetate. In contrast to constant pH simulation techniques handling proteins’ (de-)protonation, the current work deals with proton transfer within the solvent. Protex augments an OpenMM simulation object and is, therefore, straightforwardly usable with any polarizable OpenMM simulation and not restricted to protic ionic liquids. The transfer can either be instantaneously or through intermediate *λ*-states, with user-defined distances and probability criteria.

Allowing for proton transfers overcomes one of the critical limitations in classical MD simulations, *i.e.*, the formation and breaking of bonds. However, proton transfers are essential for the meaningful simulation of protic ionic liquids or other proton-exchanging solvents. In the case of the protic ionic liquid 1-methylimidazolium acetate, a slight increase in the diffusion coefficients of all species is accompanied by a significant increase in the overall conductivity *σ*(0) of the system, which is now in excellent agreement with the experimental values.

## Data Availability

The datasets presented in this study can be found in online repositories. The names of the repository/repositories and accession number(s) can be found below: https://github.com/cbc-univie/protex.
