# Supplementary material for: Protex—A Python utility for proton exchange in molecular dynamics simulations
Source: Front Chem. 2023 Feb 17;11:1140896. doi: 10.3389/fchem.2023.1140896 (PMC9981665; doi:10.3389/fchem.2023.1140896)
Supplement: Supplementary file 1 [file DataSheet1.pdf]

frontiers

Protex - A Python utility for proton exchange in molecular dynamics simulations: Electronic Supporting Information

1 PROTEX SETTINGS

Table S1 depicts the transfer numbers for the four reactions, as seen in Fig. 7. The proportion of charged species after 50 ns simulation time matches the initial 30%.

Table S1. Number of transfers for the different reactions. %IL gives the proportion of charged species in the system after the total simulation time.

Rep	$Im_1H^+ + OAc^-$	$Im_1 + HOAc$	$Im_1H^+ + Im_1$	$HOAc + OAc^-$	%IL
			s=0.25		
1	821	816	2922	863	29
2	776	775	3120	873	30
3	789	793	3139	995	31
4	837	836	3431	936	30
5	810	815	3313	963	31
Avg	807	807	3185	926	30
			s=0.4		
1	465	464	2436	226	30
2	407	410	2525	229	31
3	419	420	2378	232	30
4	442	442	2625	260	30
5	427	421	2478	221	29
Avg	432	431	2488	234	30

Protex example

Listing 1 shows an example of a minimal protex simulation. The simulation object is generated with a helper function, which returns an `OpenMM` simulation object. The specification on how to define the allowed updates and donor/acceptor atoms can be seen. The propagation of the trajectory and calling of updates are independent and can be freely combined.

```
from protex.testsystems import generate_im1h_oac_system
from protex.system import ProtexSystem, ProtexTemplates
from protex.update import NaiveMCUpdate, StateUpdate

simulation = generate_im1h_oac_system()


allowed_updates = {}
allowed_updates[frozenset(["IM1H", "OAC"])] = {"r_max": 0.16, "prob": 0.994}
```

```
allowed_updates[frozenset(["IM1", "HOAC"])] = {"r_max": 0.16, "prob": 0.098}

IM1H_IM1 = {"IM1H": {"atom_name": "H7"},
             "IM1": {"atom_name": "N2"}}
OAC_HOAC = {"OAC" : {"atom_name": "O2"},
            "HOAC": {"atom_name": "H"}}

templates = ProtexTemplates([OAC_HOAC, IM1H_IM1], allowed_updates)
ionic_liquid = ProtexSystem(simulation, templates)
update = NaiveMCUpdate(ionic_liquid)
state_update = StateUpdate(update)
for i in range(10):
    ionic_liquid.simulation.step(1000)
    state_update.update(2)
```

Listing 1: Protex example

Benchmark

Two contributions are responsible for the update process, once the calculation of the distance matrix, which is constant in time, and the actual update routine, which scales linearly with the number of molecules, which need to be updated. Tests on a GPU RTX3090 and CPU AMD Threadripper, showed, that for a typical setup with $10\,\mathrm{ps}$ simulation between updates, the calculation of the matrix takes about $11\,\%$ and the update routine $13\,\%$ of the total time. Fig. S1 illustrates the trend with increasing updates and simulation steps.

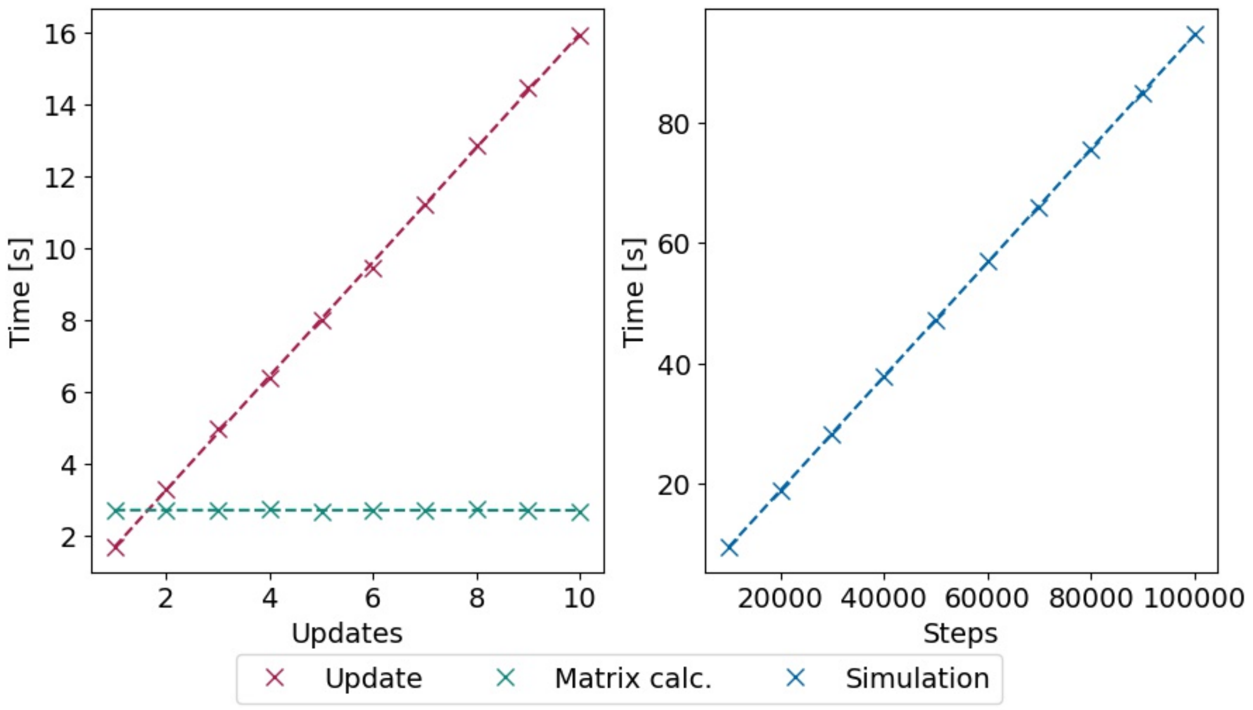

Figure S1: Benchamrk of the updates, matrix calculation and simulation steps..

2 PHYSICO-CHEMICAL PROPERTIES

2.1 Diffusion coefficients

Tables S2 and S3 show the exact numbers of the diffusion coefficients as used in Fig. 9. The average values for Im and Ac are obtained from the diffusion coefficients of the respective charged and neutral molecules weighted by their numbers in the simulation ($\approx$ 30% ionic : 70% neutral).

Table S2. Diffusion coefficients for the four species with a scaling factor of s=0.25. Im and Ac stand for combined Im_1H^+ + Im_1 and OAc^- + HOAc, respectively. The reference values for the single species are taken from Ref. 1. The experimental values are taken from Ref. 2.

No.	Im	Im_1H^+	Im_1	Ac	OAc^-	HOAc
		$[1 \times 10^{-7}\,\mathrm{cm^2\,s^{-1}}]$				
1	20.8	6.6	28.4	11.7	6.6	15.3
2	21.1	8.2	28.4	12.1	7.8	15.7
3	20.1	7.2	27.0	12.0	6.9	15.8
4	20.4	7.5	27.5	12.3	8.5	15.9
5	20.5	7.0	27.4	12.3	7.3	16.2
Avg	20.6	7.3	27.7	12.1	7.4	15.8
Ref. 1	23.9[a]	6.4	22.3	20.3[b]	6.0	13.6

[a] Exp. value for C2 Im_1H^+; [b] Exp. value for CH_3 OAc^-

Table S3. Diffusion coefficients for the four species with a scaling factor of s=0.4. Im and Ac stand for combined Im_1H^+ + Im_1 and OAc^- + HOAc, respectively. The reference values for the single species are taken from Ref. 1.

No.	Im	Im_1H^+	Im_1	Ac	OAc^-	HOAc
		$[1 \times 10^{-7}\,\mathrm{cm^2\,s^{-1}}]$				
1	14.3	6.0	19.5	9.4	5.6	11.7
2	14.6	5.7	19.7	9.7	6.0	12.1
3	15.9	5.3	21.4	10.0	5.3	12.3
4	15.7	6.2	20.8	10.0	6.0	12.9
5	14.9	5.5	20.4	9.9	6.1	12.3
Avg	15.1	5.7	20.3	9.8	5.8	12.3
Ref. 1	-	4.6	15.2	-	4.4	10.4

2.2 Collective translational dipole moment M_J

The basic problem concerning the collective translational dipole moment arises from the simulation being performed with periodic boundary conditions, *i.e.* using **folded** coordinates in the primary simulation cell. However, the analysis of the mean-squared displacement is naturally performed using the **unfolded** coordinates. During a proton transfer event, two exchanging molecules may be close together in the folded trajectory but very far apart in the unfolded trajectory. Even if the last information can be stored in memory or disk, one cannot restrict proton exchange events to molecules that are close in folded coordinates and in unfolded coordinates since this would limit the number of possible candidates. This is particularly true for long trajectories as this number would be essentially zero.

As seen in Fig. S2, the collective translational dipole moment shows big jumps at proton transfer events when analyzed the usual way. The mint curve in the inset shows this huge jump after $150\,\mathrm{ps}$, which gets eliminated after the correction.

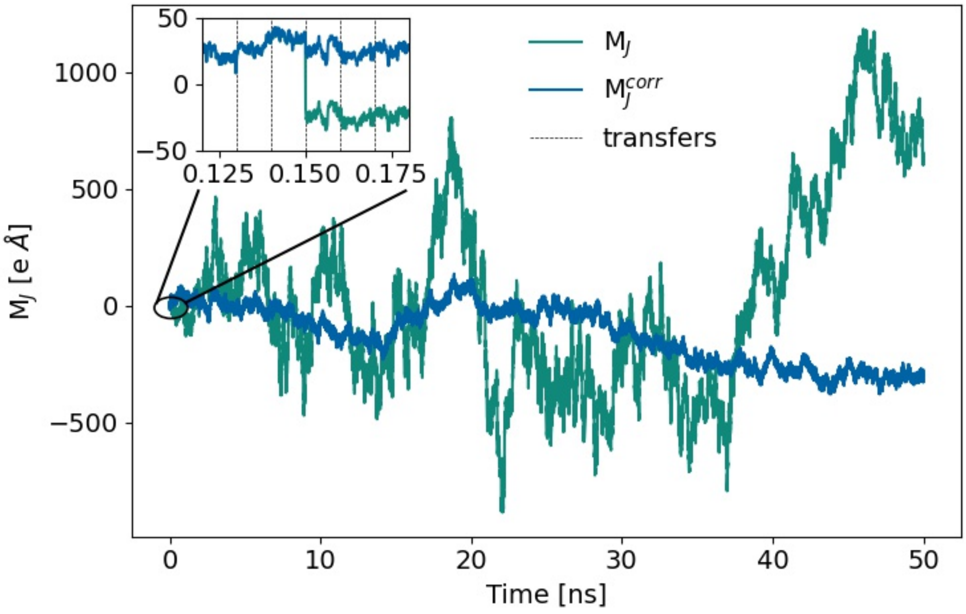

Figure S2: Collective translational dipole moment M_J in x-direction before and after correcting the timeseries.

3 VORONOI SHELL RESOLVED PMF

Table S4. Coordination numbers (N_{kl}) and Potential of mean force (PMF) for s=0.25. Numbers in brackets refer to Ref. 1

	N_{kl}			
	Im_1H^+	OAc^-	Im_1	HOAc
Im_1H^+	2.55 (2.87)	3.10 (3.32)	5.47 (5.25)	5.17 (5.01)
OAc	3.10 (3.32)	1.25 (1.21)	5.04 (4.71)	4.18 (4.01)
Im_1	2.35 (2.25)	2.17 (2.02)	6.35 (6.35)	5.63 (5.79)
HOAc	2.22 (2.15)	1.80 (1.72)	5.63 (5.79)	4.29 (4.22)
	PMF [kJ/mol]			
	Im_1H^+	OAc^-	Im_1	HOAc
Im_1H^+	-0.13 (-0.42)	-0.61 (-0.78)	0.08 (0.19)	0.22 (0.31)
OAc	-0.96 (-1.20)	1.32 (1.32)	-0.08 (0.04)	0.39 (0.45)
Im_1	0.19 (0.28)	0.39 (0.55)	-0.19 (-0.20)	0.11 (0.03)
HOAc	-0.07 (0.01)	0.45 (0.56)	-0.29 (-0.36)	0.39 (0.43)

REFERENCES

Joerg, F. and Schröder, C. (2022). Polarizable molecular dynamics simulations on the conductivity of pure 1-methylimidazolium acetate systems. *Phys. Chem. Chem. Phys.* 24, 15245–15254

Thawarkar, S., Khupse, N. D., Shinde, D. R., and Kumar, A. (2019). Understanding the behavior of mixtures of protic-aprotic and protic-protic ionic liquids: Conductivity, viscosity, diffusion coefficient and ionicity. *J. Mol. Liq.* 276, 986–994
